# ML-driven quantum dots: novel HOXB13 imaging for porokeratosis with underlying squamous dysplasia associations

**DOI:** 10.1097/MS9.0000000000005126

**Published:** 2026-05-14

**Authors:** Qamar Abbas, Muhammad Ali Johar, Muhammad Umer, Mahnoor Fatima

**Affiliations:** a Department of Medicine, King Edward Medical University, Lahore, Pakistan; b Department of Medicine, Shaheed Ziaur Rahman Medical College, Rajshahi Medical University, Bogura, Bangladesh

*To the Editor*,

Porokeratosis comprises a heterogeneous group of uncommon hereditary or acquired keratinization disorders of unknown origin, caused by abnormal clonal expansion of mutant epidermal keratinocytes influenced by genetic and exogenous factors. Several variants, including disseminated superficial porokeratosis with a locus at chromosome 18p11.3, show autosomal dominant inheritance and molecular abnormalities, such as increased p53 expression and abnormal DNA ploidy. Histologically, all variants demonstrate a cornoid lamella representing disordered keratinization, with underlying keratinocytes showing dysplasia and premature apoptosis due to altered maturation or accelerated epidermopoiesis. These histopathological and molecular features provide a basis for exploring regulatory genes, such as HOXB13 in dysplasia and malignancy^[^1^]^.

Homeobox B13 (HOXB13) is the last identified member of the homeobox gene family, functioning as a sequence-specific transcription factor that preferentially binds methylated DNA and regulates developmental pathways. It is predominantly expressed in the developing genitourinary tract, including the prostate and distal colon, and regulates terminal cell differentiation in adult tissues. Abnormal HOXB13 expression has been implicated in tumorigenesis across multiple malignancies, including breast, ovarian, prostate, gastric, and hepatocellular carcinomas, as well as lesions with squamous dysplasia^[^2^]^. Considering these oncogenic roles, machine learning (ML) has emerged as a powerful tool for computer-aided diagnosis and prognosis, particularly for early detection and grading of neoplastic and dysplastic lesions. ML systems support pathologists in assessing premalignant conditions and predicting disease progression, with deep-learning (DL) models demonstrating high diagnostic accuracy^[^3^]^.

Porokeratosis is a premalignant disorder with dysplastic keratinocytes beneath the cornoid lamella and a risk of malignant transformation, most commonly to squamous cell carcinoma and basal cell carcinoma, with an estimated incidence of 7.5–11%^[^4^]^. Malignant progression is associated with allelic loss, p53 overexpression, and involvement of proteins such as psi-3, cytokeratin, filaggrin, and involucrin, while ultraviolet radiation may act as a trigger. In light of this dysplasia-driven malignant potential, histological and molecular imaging, including HOXB13-targeted quantum dots, generates high-resolution, high-dimensional data that are well-suited for ML analysis. ML models can differentiate epithelial tissue from stroma, predict dysplastic grade, and identify patients at progression risk, highlighting the complementary value of ML-integrated imaging for improving diagnostic and prognostic precision^[^3^]^.

The clinical implementation of AI through ML and DL faces significant challenges that prevent its use as a standard method for early cancer diagnosis in pathology assessment. The research encounters obstacles due to the insufficient quality and quantity of available data, which includes both the lack of well-annotated datasets and inconsistent staining results produced by different laboratories. The “black box” nature of DL systems hinders model interpretability, while ethical challenges arise from two main issues: demographic algorithmic bias and data privacy violations under HIPAA/GDPR, as well as uncertainties surrounding misdiagnosis liability. The need for multi-center validation and prospective trials creates regulatory obstacles, requiring organizations to invest in infrastructure, which in turn prevents resource-limited areas from adopting new technologies. This situation demands collaboration among scientists from different fields to achieve proper system integration^[^5^]^.

In conclusion, porokeratosis is a premalignant disorder characterized by dysplastic keratinocytes and a risk of malignant transformation, while HOXB13 plays a key role in dysplasia and tumorigenesis. ML-integrated histological and molecular imaging offers a promising approach for early detection, grading, and risk stratification of neoplastic lesions. However, challenges such as limited validation, dataset imbalance, and technical and ethical concerns persist. Further research is needed to standardize ML methods, identify reliable biomarkers, and improve early diagnosis and clinical management in porokeratosis.

## Data Availability

Not applicable to this study.
